# A New Isolate of *Pediococcus pentosaceus* (SL001) With Antibacterial Activity Against Fish Pathogens and Potency in Facilitating the Immunity and Growth Performance of Grass Carps

**DOI:** 10.3389/fmicb.2019.01384

**Published:** 2019-06-27

**Authors:** Liang Gong, Haocheng He, Dongjie Li, Lina Cao, Tahir Ali Khan, Yanping Li, Lifei Pan, Liang Yan, Xuezhi Ding, Yunjun Sun, Youming Zhang, Ganfeng Yi, Shengbiao Hu, Liqiu Xia

**Affiliations:** State Key Laboratory of Developmental Biology of Freshwater Fishes, Hunan Provincial Key Laboratory of Microbial Molecular Biology, College of Life Science, Hunan Normal University, Changsha, China

**Keywords:** *Pediococcus pentosaceus*, antibacterial activity, grass carps, gut microbiota, fish immunity, growth rate

## Abstract

Probiotic-feeding continues to be a promising strategy to control the bacterial pathogens in aquaculture. A new *Pediococcus pentosaceus* strain (SL001) was isolated from 1000s of soil samples, which exhibited wide antimicrobial spectrum of against fish pathogens, involving *Aeromonas hydrophila*, *Aeromonas veronii*, *Aeromonas sobria*, *Edwardsiella tarda*, *Lactococcus garvieae*, and *Plesiomonas shigelloide*. The challenge test against *A. hydrophila* showed that the survival rate of SL001-supplemented group was significantly higher than that of control group (*P* < 0.05). Moreover, SL001 could stably colonize in gut of grass carp and increased mucus-secreting goblet cells and extended intestinal villi could be observed in SL001-supplemented group (*P* < 0.05). Feeding with SL001 supplemented diet could significantly enhance the growth rate (*P* < 0.05) and markedly affect gut microbiota structure of grass carps, resulting in reduced potential pathogens and increased potential probiotics. Furthermore, feeding grass carps with SL001 caused the up-regulated expression of insulin-like growth factor (IGF-1 and IGF-2) and down-regulated expression of myostatin (MSTN-1 and MSTN-2) (*P* < 0.05), which probably also account for the increased growth rate of SL001-fed group. Meanwhile, relative mRNA expression levels of immune-related genes in liver, spleen, and head kidney were analyzed in grass carps after feeding for 30 days with SL001 supplemented diets. In all three immune organs, the expression levels of immunoglobulin M (IgM) and complement 3 (C3) were significantly increased (*P* < 0.05), whereas the interleukin-8 (IL-8) was down-regulated (*P* < 0.05). Besides, whole genome sequencing revealed several probiotics properties of SL001, including organic acid synthesis, bacteriocin synthesis (coagulin), superoxide dismutase, and digestive enzymes. In conclusion, *P. pentosaceus* SL001 which could enhance immunity and promoter growth rate of grass carps, is prospective to be used as a dietary probiotic in freshwater fish aquaculture.

## Introduction

High density of culture and increase of feeding amount have caused long-term crowding stress that aggravates fish susceptibility to pathogens, and outbreaks of fish diseases have become increasingly serious (Athanassopoulou et al., 2004; DiMaggio et al., 2014; Lin et al., 2018). Usually, fishes are frequently infected by microorganisms associated with viruses, bacteria, and parasites under extremely intensive culture conditions (Song et al., 2014; Tang et al., 2018; Zhou et al., 2018). In the past many years, control strategies on fish pathogens relied mostly on the application of chemicals, such as antibiotics and disinfectants. However, extensive use of medicines has caused antibiotic resistance of pathogenic bacteria (Chandrarathna et al., 2018). Therefore, overuse of antibiotics in aquaculture should be strictly controlled, and alternative methods of controlling fish pathogens must be developed, such as using antimicrobial peptides, vaccines, and probiotics.

Recently, probiotic-feeding has been proved to be a promising strategy to control diseases. Probiotics are beneficial microorganisms introduced by implantation or colonization in specific host’s gut to reinforce the intestinal barrier, boost the immune system, or produce antimicrobial substances to suppress pathogen growth (Balcazar et al., 2006; Akhter et al., 2015). Besides, probiotics could promote digestion and enhance nutrient absorption by altering the intestinal microflora, resulting in better growth performance (Balcazar et al., 2006; Falcinelli et al., 2015). Lactic acid bacteria (LAB) and *Bacillus* species are among the most commonly used probiotic candidates (Cao et al., 2011; Banerjee and Ray, 2017; Alonso et al., 2018; Yi et al., 2018). LAB strains that have been used for fish pathogens control usually include the genera *Lactobacillus*, *Leuconostoc*, *Streptococcus*, and *Pediococcus* (Perez-Ramos et al., 2018). *Pediococcus parvulus* 2.6 produces β-glucan, which could benefit colonization of its producer in the fish gut and competition with the pathogen *Vibrio anguillarum* (Perez-Ramos et al., 2018). Feeding orange-spotted grouper with *Pediococcus pentosaceus* strain 4012 could not only enhance the growth rate of the grouper and increase the number of red blood cells, but also regulate the gene expression of the pro-/anti-inflammatory cytokines (Huang et al., 2014). Similarly, *Pediococcus acidilactici*-supplemented diet significantly increases the expression levels of alanine aminotransferase (ALT) and aspartate aminotransferase (AST) in juvenile beluga (Ghiasi et al., 2018).

In aim to screen probiotics which could antagonize bacterial fish pathogens, 100s of bacterial strains have been isolated from soil samples collected from different regions of China. Among them, a novel *P. pentosaceus* strain (SL001) exhibited excellent antibacterial activity against several important fish pathogens has been added to fish diet and its probiotics properties have been studied. The impact of SL001 feeding on the gut microbiota and growth ability of grass carps was analyzed. Subsequently, the expression of growth-related and immune-related genes of grass carps was measured after feeding with SL001-supplemented diet. Our results indicated that *P. pentosaceus* SL001 could serve as potential probiotic strain in freshwater aquaculture.

## Materials and Methods

### Bacterial Isolation, Maintenance, and Identification

Soil samples from different regions of China were collected and 10-fold serially diluted in glass tubes. 0.1 mL dilutions was spread on Man Ragosa Sharpe (MRS) agar plates and anaerobic cultured on DG250 Anaerobic workstation (DWS, United Kingdom) at 37°C for 24 h. Single colonies appeared on MRS agar plates were purified twice and stored in 25% (v/v) glycerol at -80°C. The *P. pentosaceus* SL001 was grown on MRS liquid medium at 37°C for 12 h under anaerobic condition, and then scanning electron microscopy (SEM, Hitachi Su8010, Japan) were used to observe the morphology of the bacterial cell. Biochemical characterization of SL001 was done subsequently. Genomic DNA was extracted from a SL001 using a genomic DNA extraction kit according to the manufacturer’s instructions (Sangon, China). The DNA fragments carrying 16S rRNA gene were amplified using primer pair 27F (5′ to 3′: AGAGTTTGATCCTGGCTCAG) and 1492R (5′ to 3′: CGGTTACCTTGTTACGACTT) (Wu et al., 2010; Yi et al., 2018). The polymerase chain reaction (PCR) products were purified and cloned into pMD18-T vector (TaKaRa, Japan). Single colonies were picked up and sent for sequencing. Phylogenetic tree was constructed on the basis of 16S rRNA genes by the neighbor-joining method using MEGA6.06 software and evolutionary distances were computed using the Maximum Composite Likelihood method (Tamura et al., 2004).

### Antimicrobial Activity Test

SL001 was incubated in MRS liquid medium at 37°C for 24 h under anaerobic conditions. Six fish pathogenic bacteria (Supplementary Table S1) were incubated in Luria-Bertani (LB) liquid medium at 30°C for 12 h. Overnight cultures of pathogenic bacteria were diluted to 10^7^ CFU/mL by fresh LB broth, and 200 μL dilutions were spread on LB agar plates respectively. Then, sterile oxford cups were placed on the plates dried for 30 min on vertical flow clean cabinet. 100 μL of cell-free supernatant (CFS, pH 3.48) of SL001 saturated culture was added into oxford cups. MRS and phosphate-buffered saline (PBS, need pH 3.48 with lactic acid, PL) were used as control. After 18 h of incubation at 30°C, antimicrobial activity was evaluated by measuring inhibition zones (Wang et al., 2018; Yi et al., 2018).

### Bacterial Safety Evaluation

Hepatic L8824 cell line derived from liver of grass carp was cultured at 37°C with 5% CO_2_ in DMEM (Gibico/Thermo Fisher Scientific, United States) supplemented with 10% fetal calf serum (FCS, BI, Israel) and 1% penicillin-streptomycin solution (BI, Israel). Cells were pre-incubated for 12 h in 24-well flat-bottomed plates (5 × 10^5^ cells/well) as previous described (Matsuda et al., 2017). Both SL001 CFS (10 μL) and bacterial cells (1 × 10^7^ CFU) were applied to L8824 cells and cell morphological changes were analyzed using an inverted light microscope (Leica Microsystems S.p.A, Italy). CFS of *Aeromonas hydrophila* which has been proved to be toxic to L8824 cells was used as positive control and MRS was used as negative control.

### Experimental Design

Healthy grass carps (32.1 ± 9 g) obtained from Wangcheng fish pond (Changsha, China) were transferred plastic tanks (volume 60 L) equipped with air pump in laboratory and fed with the basal diet for 1 week. Grass carps were randomly divided into six plastic tanks (38 fishes per tank) and fed twice with different diets per day at 1% of the body weight: three tanks fed with basal diet, three tanks fed with SL001 (1 × 10^9^ CFU/g) supplemented diet (Huang et al., 2014; Yi et al., 2018).

Twelve grass carps were sampled randomly from every tank after 30 days of feeding. Three grass carps were taken for immune-related and growth-related genes expression analysis. Immune organs (liver, spleen, and head kidney) and muscle were collected, sliced, and immediately frozen at -80°C until RNA isolation. Two grass carps’ gut were taken for colonization identification. Two grass carps’ gut were stored in 4% paraformaldehyde fix solution and sent for histological analysis. Five grass carps were taken for intestinal bacterial community diversity analysis by using high-throughput sequencing (Wuhan Nextomics Biotechnology, Co., Ltd., China). The rest 26 grass carps from every tank were used for challenge test against *A. hydrophila*. Liver, spleen, and head kidney from grass carps after 6 h post-infection (6 hpi) or 12 h post-infection (12 hpi) were collected for immune-related genes expression analysis.

### High-Throughput Sequence Analysis

Polymerase chain reaction amplification of the variable region (V3–V4) of tract bacterial 16S rRNA gene was carried out using primer pair 338F (5′ to 3′: ACTCCTACGGGAGGCAGCAG) and 806R (5′ to 3′: GGACTACHVGGGTWTCTAAT). Amplifications were performed in ABI GeneAmp^®^ 9700 (Applied Biosystems, United States) with initial denaturation at 95°C for 3 min, 28 cycles of 30 s at 95°C, 30 s at 58°C, 45 s at 72°C and final extension at 72°C for 10 min. PCR products were purified by using AxyPrepDNA PCR Purification Kit (Axygen, United States) and quantified using QuantiFluor^TM^-ST Fluorometer (Promega, United States). Sequencing was performed by Wuhan Nextomics Biotechnology, Co., Ltd., with Illumina MiSeq system (Illumina, United States).

Paired-end (PE) reads were procured and then assembled according to the overlap relationship (Huang et al., 2016). The operational taxonomic units (OTUs) picking was completed with a minimum pairwise identity of 97% with USEARCH software (version 7.1) (Edgar, 2010) after removing chimeras. Taxonomic analyses of sequence reads were processed using Quantitative Insights Into Microbial Ecology (QIIME) pipeline (Caporaso et al., 2010). The most abundant sequence in each OTUs was selected to perform a taxonomic classification based on the Silva database (Quast et al., 2013) using the RDP classifier (Wang et al., 2007), clustering the sequences at 97% similarity with a 0.7 confidence thresholds. Alpha diversity indices were calculated with Mothur (version v.1.30.1) (Schloss et al., 2011). ACE and Chao1 were estimated to indicate the community richness, as well as Simpson and Shannon indices were reckoned to reveal the community diversity, and Good’s coverage represented the sequencing depth. Rarefaction (Amato et al., 2013) and Shannon–Wiener curve (Wang et al., 2012) were constructed with R software. Two dimensional principal coordinates analysis (PcoA) characterized the similarities and differences of the community composition. The similarities and differences in overall bacterial community structure between each of intestine samples were detected using the UniFrac metric, and then phylogenetic tree was constructed using the unweighted pair group method with arithmetic mean (UPGMA).

### Colonization and Histology in the Intestine

Intestinal contents collected from foregut, midgut, and hindgut were washed with sterile saline (0.85%) and analyzed separately. Colonization of SL001 in intestine was measured by plating the various dilutions of intestinal contents on MRS agar plates. The appeared colonies were identified using 16S rRNA gene sequence.

Intestinal tissues collected for both SL001-supplemented group and control group (six fishes for each group) were preserved in 4% paraformaldehyde fix solution, cleared in xylene and dehydrated in ethanol solutions, and then embedded in paraffin wax. Then, tissues were sectioned to 4 μm and stained with hematoxylin and eosin (H&E) according to standard protocol before examined under a light microscope (Nikon Eclipse E100, Japan).

### Activity of Non-specific Immunological Factors Measurement

The serum samples collected at 30 days post-feeding were taken for determining the acid phosphatase (ACP) and alkaline phosphatase (AKP) activity. The measurement was performed with commercial kits from Nanjing Jiancheng Institute (Nanjing, China) according to the manufacturer’s instructions (Yi et al., 2018).

### RNA Isolation and Real-Time Quantitative PCR

Total RNA was isolated from harvested samples following instructions with modifications by using Trizol reagent (Sangon, China). Briefly, tissue (∼50 mg) was homogenized in 1 mL Trizol reagent. The solution was mixed with 0.2 mL chloroform and in an ice bath for 10 min. After centrifugation at 12000 rpm, 4°C for 10 min, the aqueous phase was transferred to a new 1.5 mL tube and added equal volume of isopropanol with subsequent ice bath for 10 min. The mixture was centrifuged, and then the precipitation was washed twice with 75% ethanol and dissolved in RNase free water. The RNA quality and quantity were assessed using agarose gel (2%) electrophoresis and the ratio of OD_260_ nm to OD_280_ nm by NanoDrop 2000 spectrophotometer (Thermo, United States) respectively.

Total RNA (1 μg) was treated with DNase and reversed transcription to synthesize cDNA by PrimeScript^TM^ RT reagent Kit with gDNA Eraser (TaKaRa, Japan) according to manufacturer’s instructions. For real-time quantitative PCR (qRT-PCR), the primer pair were designed with Primer Premier 5.0 according to the published sequences and listed in Table 1. The amplification was performed with SYBR Permix Ex Tag^TM^ GC (TaKaRa, Japan) at 7500 Real-Time PCR system instruments (Applied Biosystems, United States). The PCR cycling conditions were as follows: 2 min at 50°C and 10 min at 95°C, followed by 40 cycles of 15 s at 95°C and 1 min at 60°C. Melting curves were performed from 60 to 95°C to validate the specificity of PCRs (Palazzotto et al., 2015). The β-actin gene was used as internal control gene (Chu et al., 2018).

**Table 1:** The sequences of primers used for qRT-PCR.

Gene	Primers (5′ to 3′)	Product size (bp)	GenBank accession no.
β-act	Forward: GCTATGTGGCTCTTGACTTCG	124	M25013.1
	Reverse: GGGCACCTGAACCTCTCATT		
IgM	Forward: TGGTCATCAGGTGGCAAATAC	120	DQ417927.1
	Reverse: GCGGCTGTCTTCCATTCTT		
C3	Forward: AATACGCCATTCCTGAGGTTTC	438	AY374472.1
	Reverse: GCTTCAATGCCAACTGTCAGAC		
LSZ	Forward: TTCGACAGCAAAACAGGACA	159	FN428856.1
	Reverse: GATATGATGGCAGCAATCACAG		
IGF-1	Forward: CATTGCCCGCATCTCATC	210	EU051323.1
	Reverse: TGTCGCGGGAGTCAACG		
IGF-2	Forward: GGGTGGCATTGCGATAA	101	EF062860.1
	Reverse: CAGCCACGCTCGAAAGG		
IL-1β	Forward: TACCTTGCTTGTACCGAGTCG	107	JX014320.1
	Reverse: CAGGAGGTTGTCATGTTGGTC		
IL-8	Forward: CAATGAGTCTTAGAGGTCTGGGTG	199	JN255694.1
	Reverse: GACCTTCTTAACCCAGGGAGC		
MSTN-1	Forward: GCATGTGGTCCAGTGGGTAAT	168	KM874826.1
	Reverse: GAGCCTGTTTGAGTCGGAGTT		
MSTN-2	Forward: GATGAGCATTCCACCGAGTC	240	KM874827.1
	Reverse: CACGGTCATTGAAATACAGCAT		


### Growth Performance

To monitor the effect of SL001 on the growth of grass carps, the weight gain rate (WGR), specific growth rate (SGR), feed intake (FI), and feed conversion ratio (FCR) of grass carps were calculated as previously described (Shi et al., 2019).

$$\begin{array}{l} \text{WGR}(\%)=100\times ({\text{W}}_{\text{t}}-{\text{W}}_{0})/{\text{W}}_{0}\\ \text{SGR}(\%)=100\times (\ln{\text{W}}_{\text{t}}-\ln{\text{W}}_{0})/30\text{ days}\\ \text{FI}=\text{feed consumption}/[({\text{W}}_{0}+{\text{W}}_{\text{t}})/2\times 30\text{ days}]\\ \text{FCR}=\text{feed consumption}/({\text{W}}_{\text{t}}-{\text{W}}_{\text{0}}) \end{array}$$

W_0_ and W_t_ designates the average weights of grass carps at the start of the experiment and at the termination of the experiment respectively.

### Challenge Test

As preceding description of Yi et al. (2018). *A. hydrophila* was inoculated into LB and shaked for 12 h. Overnight culture of *A. hydrophila* was diluted to 1 × 10^7^ CFU/mL with sterile saline. Grass carps were divided into three groups: in experimental group, grass carps were fed with SL001 supplemented diet for 30 days and then intraperitoneally injected with 100 μL of *A. hydrophila* suspensions (10^6^ CFU); in challenge control group, grass carps were fed with basal diet and intraperitoneally injected with 100 μL of *A. hydrophila* suspensions (10^6^ CFU); in negative control group, grass carps were fed with basal diet and intraperitoneally injected with 100 μL sterile saline solution. Protective effect of SL001 against *A. hydrophila* was evaluated by relative percent survival (RPS) using the following formula: RPS = (1 – mortality in SL001 supplemented group/mortality in challenge control group) × 100%.

### SL001 Genome Sequencing and Analysis

Genomic DNA of SL001 was purified using magnetic beads (0.5×) and quantified using NanoDrop 2000 spectrophotometer and Qubit 4 Fluorometer (Thermo, United States). Whole genome sequencing was performed by Wuhan Nextomics Biotechnology, Co., Ltd., using the Nanopore sequencing platform. After filtering out invalid data, the assembly was performed with Canu software (v1.3) (Koren et al., 2017) and corrected using Nanopolish 0.8.4 software (Walker et al., 2014). The assembled genome was annotated with RAST sever (Overbeek et al., 2014). Genome was further analyzed with Anti-SMASH for secondary metabolite and bacteriocin biosynthesis gene clusters. The tRNA, rRNA, and CRISPR were predicted using tRNAscan-SE 1.23, RNAmmer 1.2, and CRISPRS II respectively.

### Statistical Analysis

All data were statistically analyzed using SPSS 21.0 software and presented as the mean ± SE of mean (SEM). Independent-Samples *t*-test and Least-Significant Difference test were used to calculate significant differences. *P*-value < 0.05 and < 0.01 were considered statistically significant and extremely significant, respectively.

### Ethics Statement

All sampled fish were humanely euthanized by bath immersion using an overdose of MS222 (3-Aminobenzoic acid ethyl ester methanesulfonate, Sigma, United States). This study was carried out in accordance with the recommendations of Animal Management Regulations (Directive 1988/2/CN). This study has been reviewed and approved by the ethics committee of the Hunan Normal University.

## Results

### Isolation and Characterization of a New Isolate of *P. pentosaceus*

**FIGURE 1:**
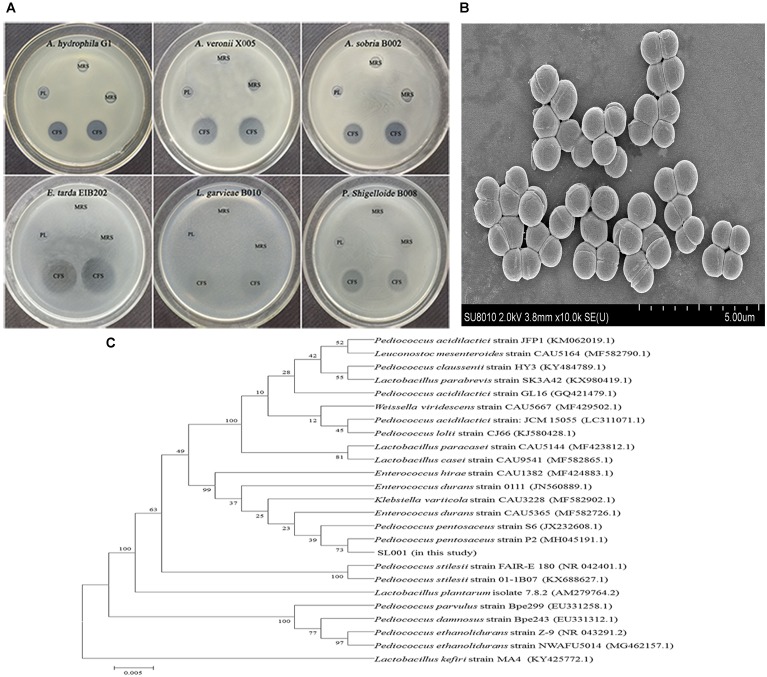
The characterization and antimicrobial activity of *Pediococcus pentosaceus* SL001. **(A)** Scanning electron microscope (SEM) of SL001. **(B)** The inhibition zones of SL001 against fish pathogens. CFS, cell-free supernatant (pH 3.48); MRS, Man Ragosa Sharpe medium; PL, phosphate-buffered saline added lactic acid (pH 3.48). **(C)** The phylogenetic tree based on 16S rRNA gene sequences inferred evolutionary relationships of strain SL001 by neighbor-joining method.

Soil samples from different regions of China were collected and subjected to screening of bacterial colonies which could inhibit the growth of bacterial fish pathogens. Among 100s of isolated bacterial strains, the isolate designated as SL001 which was isolated from soil sample collected from Dadonghai beach (located at the south coast of Sanya, China), exhibited excellent antibacterial activity against several important fish pathogens, including *Aeromonas hydrophila*, *Aeromonas veronii*, *Aeromonas sobria*, *Edwardsiella tarda*, *Lactococcus garvieae*, and *Plesiomonas shigelloide* (Figure 1A and Supplementary Figure S1B).

SL001 cells, which were spherical (0.9–1.1 μm in diameter), Gram-positive, non-spore-forming and appear in pairs or quadruples, could form opalescent and wet colonies on MRS agar plate (Figure 1B and Supplementary Figure S1A). Subsequently, SL001 was characterized on the basis of 16S rRNA gene sequence analysis, which demonstrated that SL001 clustered with *P. pentosaceus* strain P2 (GenBank Accession No. MH045191.1), with 99% similarity (Figure 1C). The results of biochemical characterization of SL001 was also consistent with that of previously reported *P. pentosaceus* strains (Supplementary Table S2) (Shukla and Goyal, 2014). Thus, SL001 was identified as a new isolate of *P. pentosaceus*.

### Biosafety Evaluation of SL001 on Fish

**FIGURE 2:**
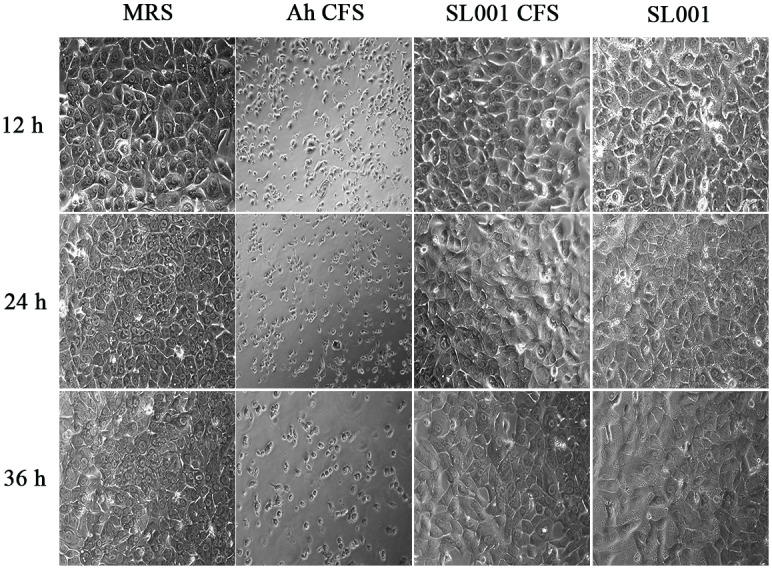
The morphology of grass carp hepatic L8824 cell line after treatment with SL001. Medium or bacterial culture applied to L8824 cells and incubated for 12, 24, and 36 h, respectively. MRS, Man Ragosa Sharpe medium (negative control); Ah CFS, cell free culture supernatant of *Aeromonas hydrophila* (positive control); SL001 CFS, cell free culture supernatant of SL001; SL001, cell pellet of SL001.

As SL001 showed remarkable potential in controlling fish pathogens, we investigated its biosafety for fish. Cell-free supernatant (CFS) and the cell pellet of SL001 saturated culture was applied to hepatic L8824 cells. After 36 h of incubation, neither CFS nor cell pellet of SL001 exhibited cytotoxicity against L8824 cells. We then evaluated the biosafety of SL001 *in vivo* by supplementing SL001 (1 × 10^8^ CFU/mL) in fish diet. After 30 days of feeding, neither mortality nor visible adverse effects was observed in grass carps (Figure 2).

### Protection of SL001 Against *A. hydrophila* Infection

**FIGURE 3:**
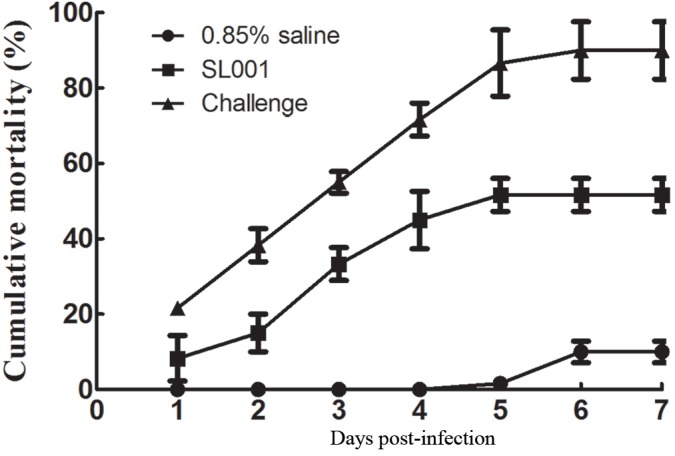
Challenge test to detect the protection of SL001 against *A. hydrophila*. The survival rates of grass carp after *A. hydrophila* infection were measured. Each value represents mean ± SEM (*n* = 3).

After 30 days of feeding with SL001-supplemented diet, grass carps were intraperitoneally challenged with *A. hydrophila* (1 × 10^6^ CFU per fish). The cumulative mortality of SL001-supplemented group after 7 days was 51.7%, which was significantly lower than that of without SL001 supplemented group (90%) (Figure 3), with a RPS value of 42.6%. Thus, it indicated that SL001 could protect grass carps from *A. hydrophila* infection.

### Effect of SL001 on the Growth of Grass Carps

**FIGURE 4:**
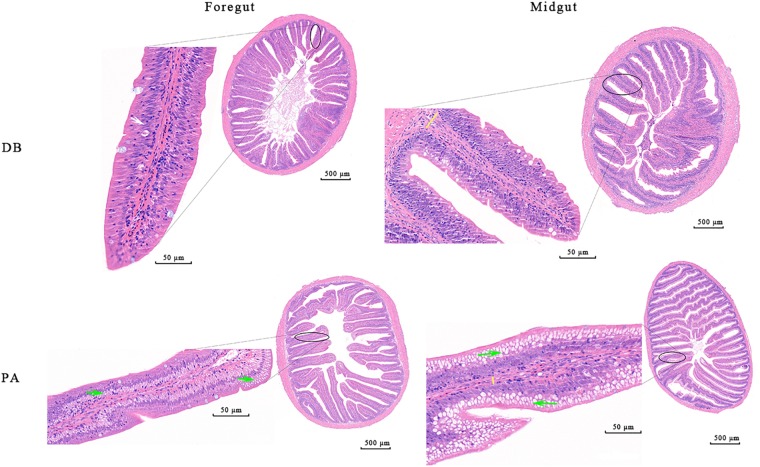
Photomicrographs of the different intestinal site of grass carp after 30 days of feeding with SL001-supplemented diet (*n* = 6, data from one fish is presented). Enterocytes (white arrow), goblet cells (red arrow), lamina propria (indicated by yellow lines) are shown in the figure. PA, SL001-supplemented group; DB, without SL001 supplemented group.

After 30 days of feeding, all the detected growth indices including weight gain rate (WGR, 15.06%), specific growth rate (SGR, 0.47%), feed intake (FI, 9.88) and feed conversion ratio (FCR, 2.35) of SL001-supplemented group were significantly increased when compared to those of control group (11.69, 0.37, 7.03, and 2.13%, respectively) (Supplementary Table S3). Histological analysis of intestine of grass carps demonstrated that normal villi, distinct lamina propria, enterocytes, and goblet cells were visible in both SL001-supplemented group and without SL001 supplemented group (*n* = 6, Figure 4). However, more mucus-secreting goblet cells and longer intestinal villi could be observed in SL001-supplemented group. This observation was more pronounced in the midgut (as the main structure of nutrient absorption), with the increase in length of villi by approximately 53% (*P* < 0.05, Supplementary Table S4), indicating that SL001 promoted nutrients absorption in grass carps. Moreover, the lamina propria in control group was wider compared with that in SL001-supplemented group (Figure 4), implying that SL001 could reduce inflammatory response in grass carps. After anaerobic cultivated, SL001 dominated in all the dilutions of intestinal contents in SL001-supplemented group and only SL001 colonies appeared on MRS agar plate in the 10^-8^ dilution, which was confirmed by 16S rRNA gene sequencing (Supplementary Figure S2).

**FIGURE 5:**
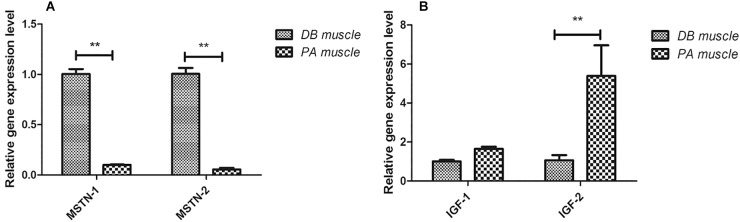
The expression levels of genes involved in muscle growth in grass carp. **(A)** Genes involved in restraining muscle growth (MSTN-1 and MSTN-2). **(B)** Genes involved in promoting muscle growth (IGF-1 and IGF-2). Values represented mean ± SEM. Each gene expression level in control group was regarded as 1. ^∗^*P* < 0.05, ^∗∗^*P* < 0.01 (*n* = 4). PA, SL001-supplemented group; DB, without SL001 supplemented group.

We subsequently examined the expression of genes involved in muscle growth regulation using qRT-PCR. In SL001-supplemented group, genes involved in restraining muscle growth (myostatin, MSTN-1, and MSTN-2) were significantly down-regulated, whereas genes related to promoting muscle growth (insulin-like growth factor, IGF-1 and IGF-2) were notably up-regulated (Figure 5).

### Influence of SL001 on Gut Microbiota of Grass Carps

**FIGURE 6:**
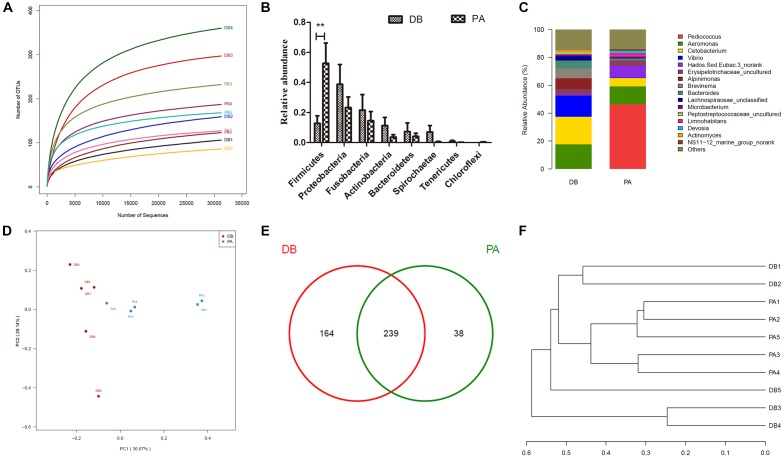
The influence of SL001 on gut microbiota of grass carps. V3–V4 region of bacterial 16S rRNA genes were sequenced and analyzed on 30 dpf grass carps. **(A)** The rarefaction curves plot of all samples. **(B)** Relative abundance in phyla level. **(C)** Stacked bar chart about the relative abundance in genus level. **(D)** Two dimensional principal coordinates analysis (PCoA) graph based on weighted UniFrac distances. **(E)** Venn diagram on shared and unique OTUs in SL001-supplemented group and without SL001 supplemented group. **(F)** The phylogenetic tree was constructed using unweighted pair group method with arithmetic mean (UPGMA). PA, SL001-supplemented group; DB, without SL001 supplemented group.

In order to evaluate the influence of SL001 on gut microbiota structure of grass carps, high-throughput sequencing was conducted to sequence 16S rRNA gene (V3–V4 region) of bacterial community from intestine of grass carps at 30 days post-feeding (dpf). A total of 431493 valid sequences and 441 OTUs were obtained. The rarefaction curves reached a saturation phase at approximately 360 OUTs, which indicated that the sequencing data were reasonable and could reliably describe the full microbial diversity (Figure 6A). This conclusion was further verified by the Good’s coverage (Table 2) and Shannon–Wiener curve (Supplementary Figure S3A). The LDA Effect Size (LEf Se) also revealed that the abundance of gut microbiota between SL001-supplemented group and without SL001-supplemented group exhibited significant differences. *Pediococcus*, Lactobacillaceae, Lactobacillales and bacilli were the over-represented taxon in SL001-supplemented group (Supplementary Figure S3B). Alpha diversity indices showed a higher microbial diversity and lower community richness in SL001-supplemented group compared to without SL001-supplemented group (Table 2). PCoA plots based on weighted UniFrac distances were used to evaluate bacterial community composition, which showed a clear clustering pattern that samples were largely partitioned based on the SL001-feeding (Figure 6D). The existence of statistically significant differences in both abundance and community composition between SL001-supplemented group and without SL001-supplemented group (Figure 6F) indicated that SL001 could affect, to a great extent, gut microbiota of grass carps.

**Table 2:** Alpha diversity metrics of Ace, Chao1, Shannon’s diversity, Simpson, and Good’s coverage of grass carps of 30 dpf (*n* = 5).

	Ace	Chao1	Shannon	Simpson	Good’s coverage
DB	170.5 ± 3.2	183.0 ± 4.2	0.99 ± 0.08	0.161 ± 0.025	0.998833 ± 0.000068
PA	147.5 ± 2.0	148.5 ± 2.1	1.36 ± 0.18	0.666 ± 0.007	0.999105 ± 0.000135


All sequences were classified into 20 phyla and 285 genera. As a whole, the phyla with relative abundance of above 0.1% were clearly observed in the bar graph (Figure 6B). In general, the dominant phyla (abundance > 10%) in both groups were Firmicutes, Proteobacteria, and Fusobacteria. However, the relative abundance of Firmicutes in SL001-supplemented group was around five times higher than that in without SL001-supplemented group (Figure 6B). In genus level, *Pediococcus*, which was the dominant genus in SL001-supplemented group (46.3% of all reads), could hardly be detected in non-SL001-supplemented group (0.007% of all reads), indicating that SL001 could inhabit the gut of grass carps (*P* < 0.05, Figure 6C). Importantly, two fish pathogens, namely *Aeromonas* and *Vibrio* showed a higher abundance in without SL001-supplemented group (17.6 and 15.1%, respectively) than that in SL001-supplemented group (12.9 and 0.3%, respectively) (Figure 6C and Supplementary Figure S3C). The shared microbiome, discovered in both groups, was identified and comprised 239 OTUs; 38 OTUs were unique in SL001-supplemented group and 164 OTUs were unique in without SL001 supplemented group (Figure 6E).

### Expression of Immune-Related Genes After Treatment With SL001

**FIGURE 7:**
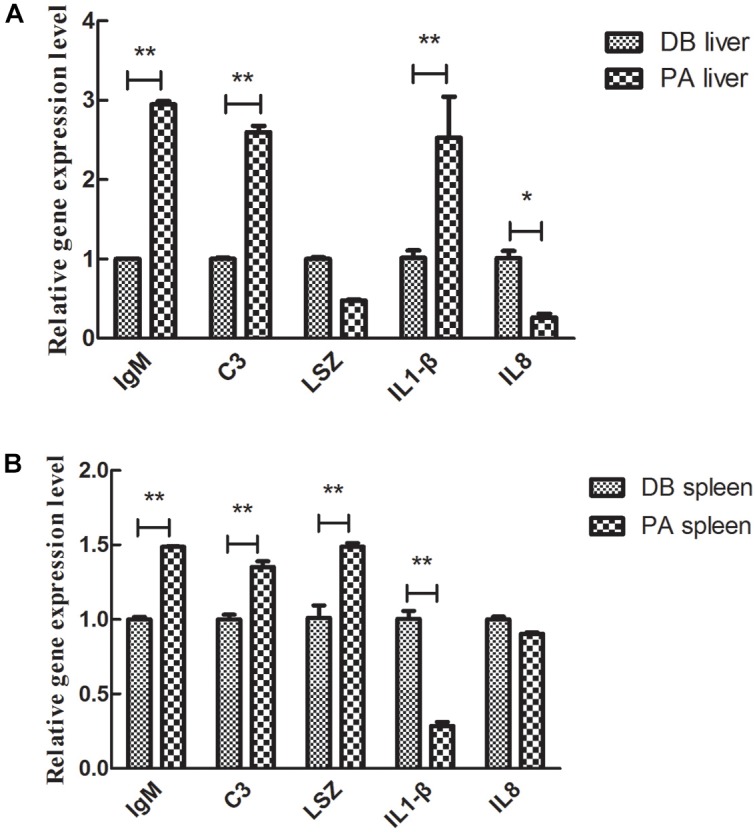
The effect of SL001-feeding on the gene expression of immune-related cytokines in the grass carp. The gene expression levels of immune-related cytokines in **(A)** liver and **(B)** spleen were measured by qRT-PCR. Values represented mean ± SEM. Each gene expression level in control group was regarded as 1. ^∗^*P* < 0.05, ^∗∗^*P* < 0.01 (*n* = 4). PA, SL001-supplemented group; DB, without SL001 supplemented group.

The expression levels of genes involved in fish immunity were determined. After 30 days of feeding with SL001-supplemented diet or basal diet. In the liver, the expression levels of all detected genes but lysozyme (LSZ) gene showed significant differences between SL001-supplemented group and without SL001-supplemented group (Figure 7A). The gene expression levels of immunoglobulin M (IgM), complement 3 (C3), and interleukin-1β (IL-1β) in SL001-supplemented group elevated 3-, 2.6-, and 2.5-fold respectively (Figure 7A). The expression level of interleukin-8 (IL-8) decreased and maintained 1/3 level of without SL001-supplemented group (Figure 7A). In spleen, the gene expression of IgM, C3, and LSZ were significantly upregulated by SL001 after 30 days of feeding, with 1.5-, 1.4-, and 1.5-fold increases respectively (Figure 7B). By contrast, the gene expression levels of both genes from the interleukin (IL) family were down-regulated (Figure 7B), among which IL-1β gene expression showed a considerable decrease in SL001-supplemented group (Figure 7B). The down-regulation of two pro-inflammatory cytokine (IL-1β, IL-8) indicated the reduction of host’s inflammatory response, and up-regulated expression of IgM, C3, and LSZ suggested that both specific and non-specific immunity of grass carps were improved in SL001-supplemented group.

**FIGURE 8:**
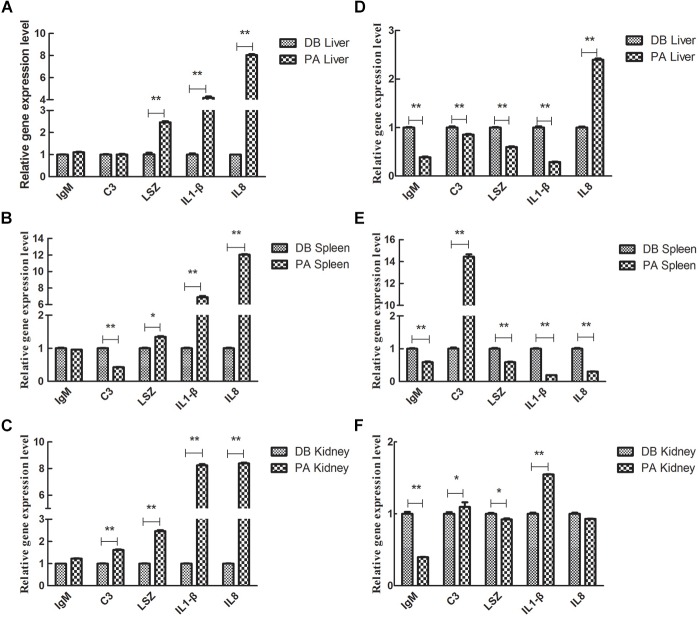
The relative gene expression level of immune-related cytokines in grass carps in challenge test. The grass carp were fed with or without SL001-supplemented diet for 30 days and then infected with *A. hydrophila*. The gene expression levels of immune-related cytokines in liver **(A,D)** and spleen **(B,E)** and head-kidney **(C,F)** were analyzed **(A–C)** were at 6 dpi **(D–F)** were 12 dpi. Values represented mean ± SEM. Each gene expression level in control group was regarded as 1. ^∗^*P* < 0.05, ^∗∗^*P* < 0.01 (*n* = 4). PA, SL001-supplemented group; DB, without SL001 supplemented group.

We further analyzed the influence of 30 days-feeding with SL001-supplemented diet on the expression of immune genes in grass carps post *A. hydrophila* infection. After feeding grass carps with SL001-supplemented diet for 30 days, the grass carps were challenged with 1 × 10^6^ CFU/mL *A. hydrophila*, and genes involved in fish immunity were determined. In the liver, the gene expression levels of LSZ, IL-1β, and IL-8 in the SL001-supplemented group were significantly higher than those of the non-supplement group at 6 hpi (Figure 8A). IL-8 gene expression in the SL001-supplemented group at 12 hpi was significantly higher than that of the non-supplement group. However, the gene expression levels of IgM, C3, LSZ, and IL-1β in the SL001-fed group at 12 hpi were prominently down-regulated compared with those of the control group (Figure 8D). In the spleen, the C3 gene expression levels in the SL001-supplemented grass carps exhibited notable decrease at 6 hpi, and then increased remarkably at 12 hpi (Figure 8B,E). By contrast, the gene expression levels of LSZ, IL-1β, and IL-8 in the SL001-fed grass carps were significantly higher at 6 hpi and became considerably lower at 12 hpi (Figure 8B,E). In addition, the expression of IgM in the SL001-supplemented group was evidently down-regulated at 12 hpi (Figure 8E). In the head-kidney, the gene expression levels of C3 and IL-1β in the SL001-fed grass carps at 6 and 12 hpi were higher than those of the non-SL001-fed grass carps (Figure 8C,F). LSZ gene expression in SL001-fed grass carps was notably upregulated at 6 hpi and then significantly down-regulated at 12 hpi (Figure 8C,F). The gene expression levels of IgM in the SL001-fed grass carps were evidently lower at 12 hpi, compared to that of the control grass carps (Figure 8F). In the early stage of infection (6 hpi), pro-inflammatory cytokines (IL1β, IL8) and LSZ were significantly up-regulated in the immune organ of grass carp fed with SL001-diets, which was beneficial to the body against pathogens. In the late infected stage (12 hpi), the higher expression level of C3 indicated that could eliminate pathogens, because of SL001 stimulating the body to improve its immunity.

**FIGURE 9:**
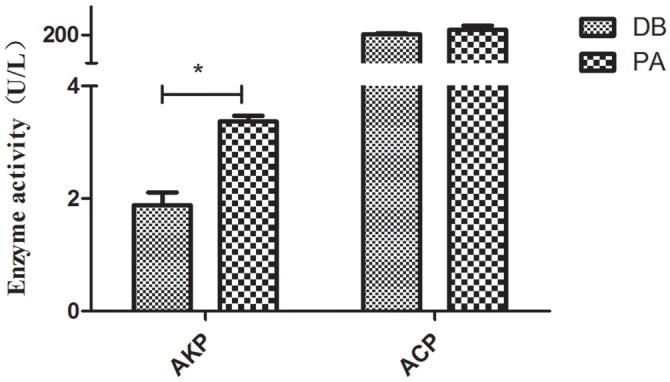
The enzyme activity of non-specific immunological factors from serum of grass carp. Values represented mean ± SEM. ^∗^*P* < 0.05, ^∗∗^*P* < 0.01 (*n* = 3). PA, SL001-supplemented group; DB, without SL001 supplemented group.

Besides, alkaline phosphatase (AKP) activity in SL001-supplemented groups was significantly increased compared with the control group after 30 dpf (*P* < 0.05). The activity of ACP was also increased by SL001 supplemented diets albeit not statistically significant (Figure 9).

### Whole Genome of SL001

**FIGURE 10:**
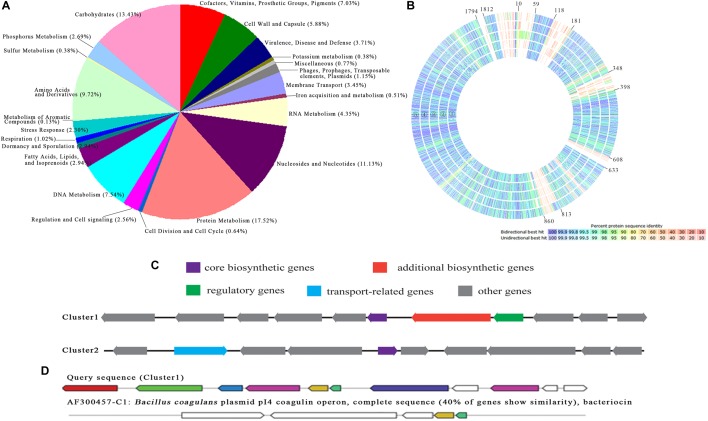
The annotation and comparison of *P. pentosaceus* SL001 genome. **(A)** The distribution of predicted CDSs of SL001 in different categories of metabolic function. **(B)** A full genome comparison analysis of *P. pentosaceus* SL001 with *P. pentosaceus* strains. 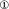 *P. pentosaceus* wikim20; 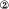 *P. pentosaceus* SL4; 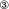 *P. pentosaceus* SRCM100892; 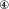 *P. pentosaceus* KCCM 40703; 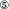 *P. pentosaceus* ATCC 25745. **(C)** Bacteriocin synthesis gene clusters identified in the genome of *P. pentosaceus* SL001. **(D)** The homology analysis of the gene cluster 1 in genome of SL001 and coagulin biosynthetic gene cluster from *B. coagulans*.

The whole genome of *P. pentosaceus* strain SL001 was sequenced using third-generation DNA sequencing, which generated 1,842,476 bp longest contig and 76,699 bp shortest contig, resulting in a genome assembly with total size of 1,917,175 bp (GenBank accession no. CP039378 and CP039379) and G+C content 37.4%. 1,905 coding DNA sequences (CDSs), 72 RNA (including 57 tRNAs and 15 rRNAs), and 22 CRISPRs were obtained by the method of NCBI prokaryotic genome annotation pipeline. Online software RAST was used to annotate the genome, the results of which showed that 41% of the annotated CDS were assorted to the subsystem (Figure 10A). Among the CDS, protein metabolism was the most-enriched metabolic category. In addition, a full-genome comparison analysis of *P. pentosaceus* SL001 with already-sequenced *P. pentosaceus* strains showed significant differences for carbohydrates and DNA metabolism (in genes 10–59, 118–181, 348–398, 608–633, 813–860, and 1794-1812) (Figure 10B).

The SL001 genome includes numerous genes involved in organic acid synthesis [e.g., *gar*R (ORF540), *pgk* (ORF1671), *glxk* (ORF1746), *als*D (ORF490), and *ilv*B (ORF491)], as well as superoxide dismutase [SOD, e.g., *per*R (ORF558) and *npx* (ORF643, ORF1158, ORF1182)], and digestive enzymes [e.g., *pep*E (ORF865, ORF1344, ORF1780) and *IV*86 (ORF1278)]. In addition, two bacteriocin synthesis gene clusters were present in SL001 genome (Figure 10C). DNA sequence of cluster 1 (833557 to 842637) shares 40% identity with coagulin biosynthetic gene cluster from *Bacillus coagulans* plasmid pI4 (Figure 10D), while cluster 2 (853776 to 863958) was an unknown bacteriocin that possibility synthesize new antimicrobial compound (Figure 10C).

## Discussion

*P. pentosaceus* which can antagonize against several important pathogens (*Listeria*, *Enterococcus faecium*, *Pseudomonas aeruginosa*, and *Klebsiella pneumoniae*) has been applied in the food industry to control foodborne pathogenic bacteria (Bajpai et al., 2016; Meira et al., 2017; Sriphochanart and Skolpap, 2018), as well as probiotic in animals and humans (Todorov and Dicks, 2009; Bajpai et al., 2016; Cavicchioli et al., 2017). Although *P. pentosaceus* had a great potential as a probiotic in marine fishes, its probiotics property in freshwater fish has seldom been touched (Xing et al., 2013; Huang et al., 2014). In this study, a new *P. pentosaceus* strain SL001 was isolated from soil sample of Dadonghai beach in Hainan Province. SL001 exhibited broad-spectrum antibacterial activity against fish pathogens. qRT-PCR analysis demonstrated that feeding with SL001 could not only protect grass carps from *A. hydrophila* infection, but also promote growth rate. In SL001-supplemented group, more mucus-secreting goblet cells and longer intestinal villi were observed and up-regulated expression of insulin-like growth factor (IGF-1 and IGF-2) and down-regulated expression of myostatin (MSTN-1 and MSTN-2). Relative mRNA expression levels of immune-related genes in liver, spleen and head kidney were analyzed. Genome sequencing of SL001 has been performed to reveal its probiotics properties.

Fish probiotics with good colonization in the gut are an important element in growth and immune regulation (Hai, 2015; Lazado et al., 2015). Here, we demonstrated that SL001 could not only stably colonize in the intestine of grass carps but also prominently affect gut microbiota structure. The composition of gut microbiota was tightly associated with the growth of grass carps, the higher proportion relative abundance of Firmicutes over Bacteroidetes the faster growth of fish (Li et al., 2013). The relative abundance of Firmicutes in SL001-supplemented group was around five times higher than that in control group, which resulted in the increased proportion relative abundance of Firmicutes over Bacteroidetes. The influence of SL001 on the gut microbiota structure of grass carps could be responsible for the increased growth rate. Intestine goblet cells are responsible for the production of the protective mucus layers by synthesizing and secreting mucins. Goblet cells are polarized mucus-secreting cells, which could serve as a source of several digestive enzymes, including lipase, amylase, and trypsin (O’Neill et al., 2011). Efficient digestion and absorption of nutrients by the intestine require a very large apical surface area, a feature that is enhanced by the presence of villi (Freddo et al., 2016). We inferred that elevated mucus-secreting goblet cells and elongated intestinal villi in the SL001-fed group were also key factors for increased growth rate of grass carps.

Commensal probiotics can not only promoter growth of host but also provide protection from pathogenic bacteria, by creating inhibitory compounds, competing for adhesion sites or modulating immune responses (Perez et al., 2010). It was reported that *P. fluorescens* strain AH2 was able to inhibit the growth of *V. anguillarum* and increased the survival rate of infected fish (Gram et al., 1999). *Bacillus* P64 showed both probiotic and immuno-stimulatory features, which could promote growth and improve immune factors activity of host (Gullian et al., 2004). According to previous reports (Isolauri et al., 2002; Ramesh et al., 2017; Meng et al., 2019), IgM, a earliest immune molecule, was produced in fish-specific immune response. C3, LSZ, IL1β, and IL-8 played an important role in fish infection prevention and inflammatory response, which could quickly counterwork against invading pathogens and participate in non-specific immunity of fish. In our study, up-regulated expression of IgM, C3, and LSZ encoding genes and down-regulated expression of pro-inflammatory cytokines (IL-1β and IL-8) in SL001-supplemented group were detected. We speculated that SL001 which could produce prebiotics (exopolysaccharides, SOD, etc.) caused the downregulation of two pro-inflammatory cytokine genes and activated the host’s immune system, so that the significant upregulation of LSZ and C3 (involved in non-specific immunity) and the IgM (involved in specific immunity). Previous studies also showed that probiotic *Bacillus aerophilus* KADR3 significantly enhanced the levels of serum lysozyme and serum IgM and elevated the activity of the complement pathway, while *P. pentosaceus*, *L. plantarum*, and *L. brevis* reduced the expression levels of IL-1β and IL-8 (Isolauri et al., 2002; Huang et al., 2014; Ramesh et al., 2017). In addition, ACP localized within lysosomes, is a marker for determining whether the macrophages are activated (Song et al., 2006). AKP is an extracellular enzyme, which could hydrolyzes phosphate group within various organic compounds like proteins, nucleic acid (El-Ebiarie, 2012). ACP and AKP as important markers were usually used to evaluate the function of probiotic bacteria in aquaculture. Yi reported that the levels of ACP and AKP of *Carassius auratus* fed with *B. velezensis* JW supplemented diets were higher compared to the control group (Yi et al., 2018), which was verified in our study. Besides, the lamina propria in control group was wider than that in SL001-supplemented group. Vasanth et al. (2015) reported that the width of lamina propria could indirectly reflect the host’s health, in which a smaller width indicated no onset of inflammation and reflected healthier host. After challenged against *A. hydrophila*, LSZ was significantly up-regulated and then down-regulated in all three immune organs, which are consistent with Zhang’s results (Zhang et al., 2018). IL-1β and IL-8 are an immune-associated cytokines that play important roles in innate immune responses (Harada et al., 1994). Feeding grass carps with SL001-supplemenged diet resulted in a significant increase in expression of IL1β and IL8 at 6 h after infection.

By analyzing the genome of SL001, we obtained some clues about its probiotics properties. Genes related to organic acids, bacteriocins, SOD, and digestive enzymes which were thought to be linked to disease-resistance, immune response, and growth were identified in the genome of SL001. It was well-known that organic acid and bacteriocin could inhibit the growth of pathogenic bacteria and regulated the micro-ecological balance of the gastrointestinal and reproductive tract of animals (Muhialdin et al., 2018). Previous report demonstrated that the SOD enhanced humoral and cellular immunity in animals and improved disease resistance (Picchietti et al., 2009). In addition, the digestive enzymes from SL001 secretion promoted the digestion and absorption of nutrients by fish, thereby promoting fish growth (Suzer et al., 2008). Besides, two putative gene clusters responsible for bacteriocins synthesis were present in SL001 genome. One gene cluster was predicated to produce coagulin, which was reported to exhibite antimicrobial activity against pathogenic bacteria (Hyronimus et al., 1998). Coagulin, a new member of the pediocin-like family protein, was firstly discovered in *B. coagulans*. Afterward, coagulin was also identified in *P. pentosaceus* MZF16 (Zommiti et al., 2018). The difference between coagulin and pediocin was only a single amino acid residue at their C terminus (Le Marrec et al., 2000).

## Conclusion

Our work showed that feeding with SL001-supplemented diet can promote growth on grass carps and enhance its immune. We envisioned that this can be expanded to other freshwater fish aquaculture. It would undoubtedly provide an important strain for freshwater fish aquaculture and broaden the application of *P. pentosaceus*. Encapsulation of *P. pentosaceus* Li05 in an alginate-gelatin microgels significantly enhanced their viability under different conditions (Yao et al., 2018). Formulated and encapsulated probiotics can improve their survival and colonization and avoid loss of probiotics owing to the reduced probiotic activity during food storage and gastrointestinal transit during feeding. We will then focus on the microecological preparation *P. pentosaceus* for its practical application in future.

## Data Availability

This manuscript contains previously unpublished data. The name of the repository and accession number are not available.

## Author Contributions

LG and LP performed the bacterial strain isolation and identification. LC, HH, and LY performed the analysis of antimicrobial activity and determination of non-specific immunological factors activity. DL, LC, HH, and XD performed the cellular culture, challenge test, and weight analysis. YL and YS performed the collection of gut, liver, spleen, kidney, and muscle. GY performed the H&E staining. LG, HH, DL, and SH performed the Total RNA isolation and qRT-PCR analysis. LG, YZ, and LX analyzed the data of high-throughput sequence and genome. LG, HH, SH, and LX designed the study and wrote the draft of manuscript. TK, SH, and LX corrected the manuscript. All authors discussed the results and approved the final manuscript.

## Conflict of Interest Statement

The authors declare that the research was conducted in the absence of any commercial or financial relationships that could be construed as a potential conflict of interest.
