# Supplementary material for: A New Isolate of Pediococcus pentosaceus (SL001) With Antibacterial Activity Against Fish Pathogens and Potency in Facilitating the Immunity and Growth Performance of Grass Carps
Source: Front Microbiol. 2019 Jun 27;10:1384. doi: 10.3389/fmicb.2019.01384 (PMC6610308; doi:10.3389/fmicb.2019.01384)
Supplement: Supplementary file 1 [file Data_Sheet_1.docx]

***Supplementary Material***

**A New Isolate of *Pediococcus pentosaceus* (SL001) with Antibacterial Activity against Fish Pathogens and Potency in Facilitating the Immunity and Growth Performance for Grass Carp**

Liang Gong#, Haocheng He#, Dongjie Li, Lina Cao, Tahir Ali Khan, Yanping Li, Lifei Pan, Liang Yan, Xuezhi Ding, Yunjun Sun, Youming Zhang, Ganfeng Yi, Shengbiao Hu^*^, Liqiu Xia ^*^

State Key Laboratory of Developmental Biology of Freshwater Fish, Hunan Provincial Key Laboratory of Microbial Molecular Biology, College of Life Science, Hunan Normal University, Changsha 410081, People’s Republic of China.

#These authors contributed equally to this work.

***Corresponding author:**

Shengbiao Hu

shengbiaohu@hunnu.edu.cn

Liqiu Xia

xialq@hunnu.edu.cn.

Tel/Fax: 86-0731-88872905

Table S1 The fish pathogenic bacteria used in this study

| strain | Description | Reference or source |
| --- | --- | --- |
| *Aeromonas hydrophila* G1 | Isolate from a disease outbreak on grass carp | Our laboratory |
| *Aeromonas veronii* X005 | Isolate from a disease outbreak on grass carp | Our laboratory |
| *Aeromonas sobria* B002 | Isolate from a disease outbreak on grass carp | Our laboratory |
| *Edwardsiella tarda* EIB202 | Wild-type strain, CCTCC M208068, Colr, Cmr | Prof. Zhang Y.X.’s gift |
| *Lactococcus garvieae* B010 | Isolate from a disease outbreak on grass carp | Our laboratory |
| *Plesiomonas Shigelloide* B008 | Isolate from a disease outbreak on grass carp | Our laboratory |

Table S2 Fermentation of different carbohydrates by *P. pentosaceus* SL001

| Carbohydrate | Isolate SL001 | *P. pentosaceus* SPA |
| --- | --- | --- |
| Dextrose | +++ | +++ |
| Fructose | +++ | +++ |
| Sorbitol | - | nd |
| Trehalose | ++ | +++ |
| Mannitol | - | - |
| Maltose | ++ | +++ |
| Arabinose | ++ | nd |
| Xylose | - | + |
| Lactose | + | ++ |
| Rhamnose | + | ++ |

(+++) Strongly positive; (++) fairly positive; (+) weakly positive; (-) negative; nd not determined

Table S3 Effect of SL001 on growth performance of grass carp (n=20)

| Groups | W_0_ (g) | W_t_ (g) | WGR (%) | SGR (%) | FI | FCR |
| --- | --- | --- | --- | --- | --- | --- |
| PA | 35.16±10.54 | 40.45±12.51 | 15.06±0.16 | 0.47±0.005 | 9.88±0.08 | 2.35±0.02 |
| DB | 29.13±7.91 | 32.53±9.03 | 11.69±1.20 | 0.37±0.036 | 7.03±0.08 | 2.13±0.02 |

Table S4 The statistical evaluation of villi length in different intestinal site

| Intestinal site | DB | PA |
| --- | --- | --- |
| Foregut | 0.67±0.05 mm | 0.82±0.09 mm |
| Midgut | 0.60±0.03 mm | 0.92±0.11 mm * |


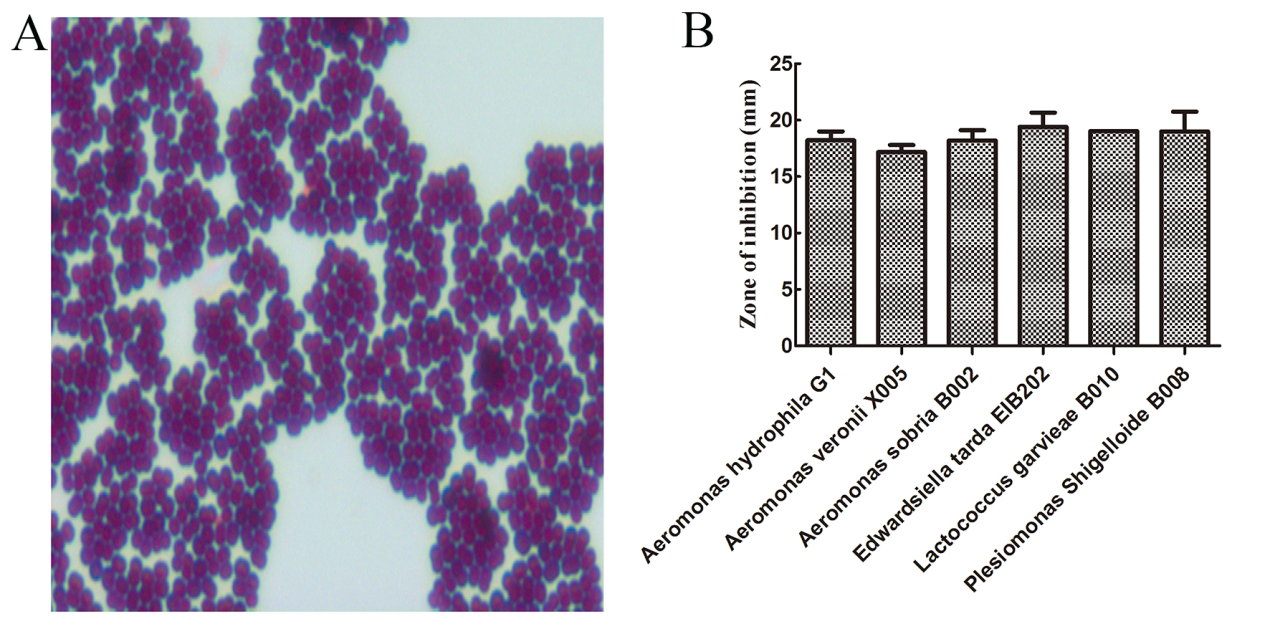


**FIGURE. S1** **(A)** The gram-staining of SL001. (B) The inhibition zone diameter of *P. pentosaceus* strain SL001 against six fish pathogenic bacteria.


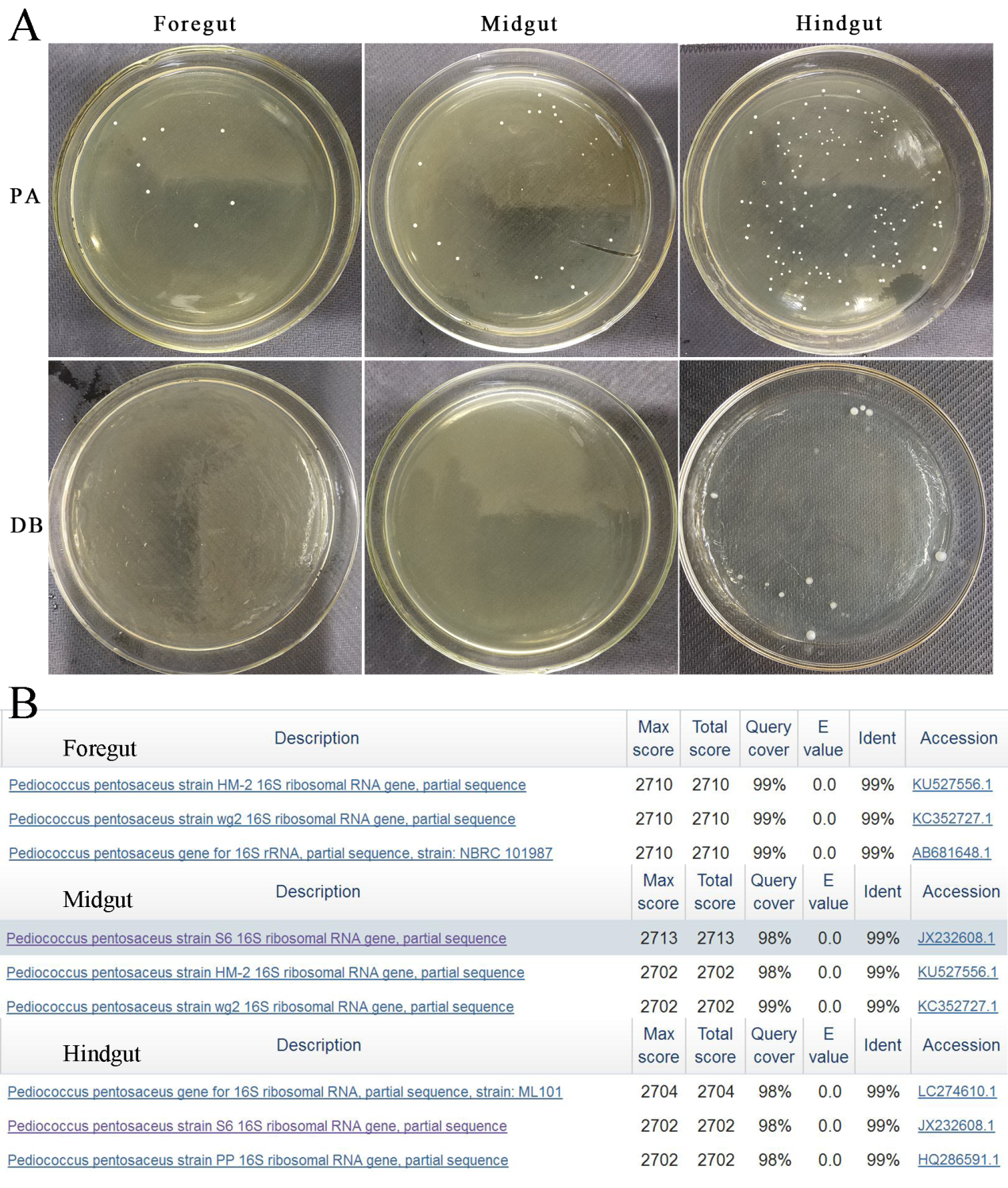


**FIGURE S2** Colonization analysis of SL001 in grass carps gut. **(A)** The strains were anaerobic screened from intestinal contents of grass carps after feeding with SL001 supplemented diets or not for 30 days. **(B)** The isolated strains from SL001-fed grass carps were identified by 16S rRNA gene sequence, and the strains in control group (DB) were identified as not a *P. pentosaceus*.


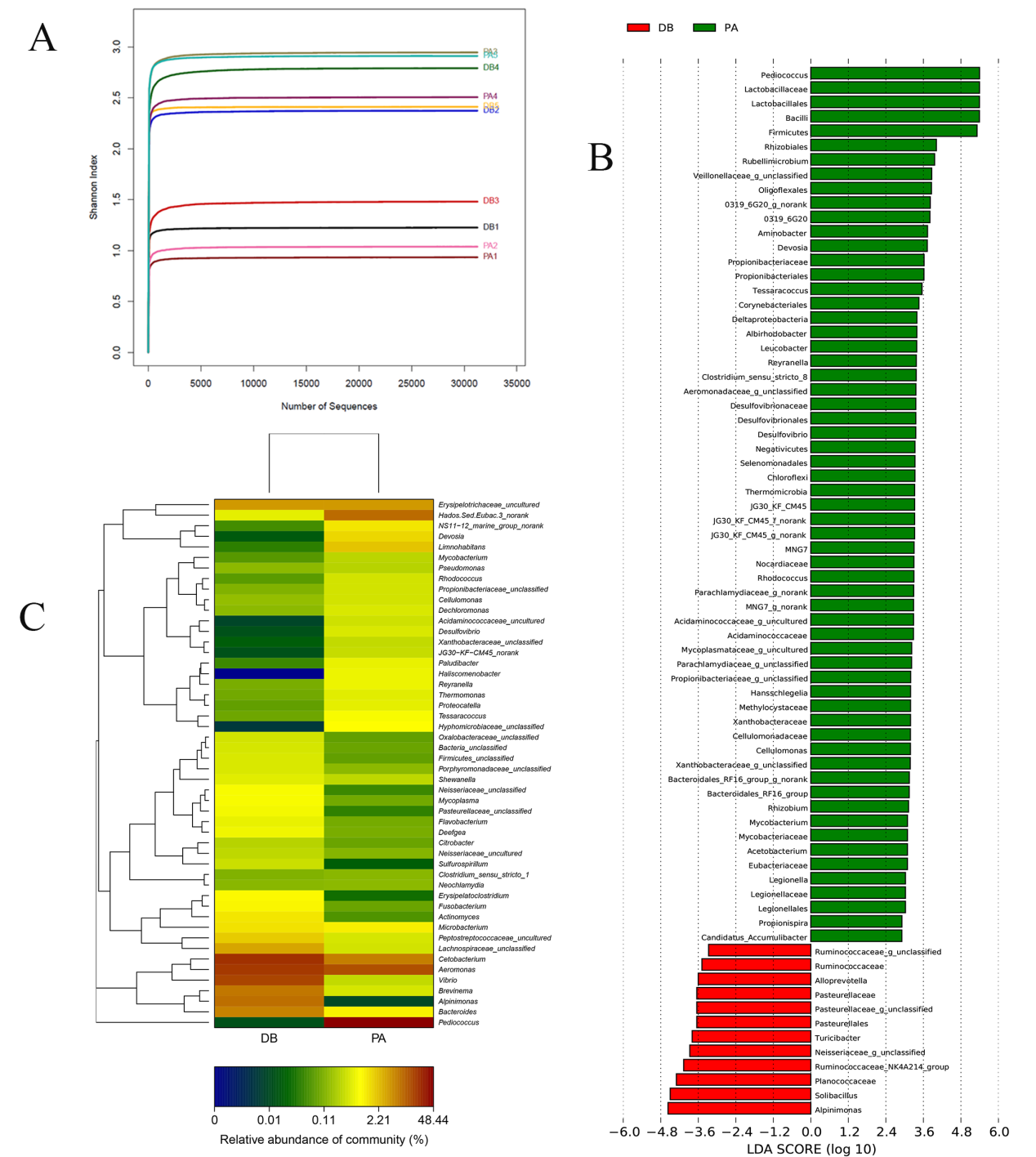


**FIGURE S3** **(A)** The shannon-wiener curve were constructed, indicating a reasonable number of individual samples. **(B)** LEfSe analysis in DB group and PA group, revealed that bacterium played an important role in each group. **(C)** The heatmap of relative abundance rankings at the top 50 in genus level.
